# Modeling and Optimization of the Multiobjective Stochastic Joint Replenishment and Delivery Problem under Supply Chain Environment

**DOI:** 10.1155/2013/916057

**Published:** 2013-10-31

**Authors:** Lin Wang, Hui Qu, Shan Liu, Cai-xia Dun

**Affiliations:** ^1^School of Management, Huazhong University of Science and Technology, Wuhan 430074, China; ^2^Economics and Management School, Wuhan University, Wuhan 430072, China

## Abstract

As a practical inventory and transportation problem, it is important to synthesize several objectives for the joint replenishment and delivery (JRD) decision. In this paper, a new multiobjective stochastic JRD (MSJRD) of the one-warehouse and *n*-retailer systems considering the balance of service level and total cost simultaneously is proposed. The goal of this problem is to decide the reasonable replenishment interval, safety stock factor, and traveling routing. Secondly, two approaches are designed to handle this complex multi-objective optimization problem. Linear programming (LP) approach converts the multi-objective to single objective, while a multi-objective evolution algorithm (MOEA) solves a multi-objective problem directly. Thirdly, three intelligent optimization algorithms, differential evolution algorithm (DE), hybrid DE (HDE), and genetic algorithm (GA), are utilized in LP-based and MOEA-based approaches. Results of the MSJRD with LP-based and MOEA-based approaches are compared by a contrastive numerical example. To analyses the nondominated solution of MOEA, a metric is also used to measure the distribution of the last generation solution. Results show that HDE outperforms DE and GA whenever LP or MOEA is adopted.

## 1. Introduction

The joint replenishment problem (JRP) is a practical inventory problem of a group of products that can be jointly ordered from a single supplier (Goyal [1]; Wang et al. [2]), which can help save the ordering costs and inventory holding costs. According to the characteristic of demand, the existing study of JRPs can be divided into two categories: (1) constant demand; (2) stochastic or dynamic demand. An extensive literature review is available in Khouja and Goyal [3] and Narayanan et al. [4]. Many scholars also discussed more realistic JRPs (J.-M. Chen and T.-H. Chen [5]; Axsäter et al. [6]; Hsu [7]; Abdul-Jalbar et al. [8]).

Many companies have realized that a joint replenishment and delivery scheduling (JRD) policy can result in considerable cost savings. But the literature on the JRDs under supply chain environment is limited. A stochastic JRD of the one-warehouse, *n*-retailer system has been formulated (Qu et al. [9]). Wang et al. [10] studied the same JRD but reduced the decision variables using specific mathematical method and provided a new differential evolution algorithm. Sindhuchao et al. [11] studied the coordinated inventory and transportation decisions with the vehicle capacity limitation in an inbound commodity collection system. Chan et al. [12] addressed issues in scheduling of the multi-item, multibuyer, and single supplier system. Cha et al. [13] handled the JRD of the one-warehouse and *n*-retailer system in which the warehouse supplies items from the supplier and delivers them to retailers. Moon et al. [14] modified the model of [13] by utilizing a consolidated freight delivery policies. Wang et al. [2] extended the JRD model of Cha et al. [13] under fuzzy environment and used the widely used signed distance method to ranking fuzzy numbers. Wang et al. [15] studied the JRD with deterministic demand and fuzzy cost using the graded mean integration representation and centroid approaches to defuzzify the total costs. A common limitation in all the literature studies mentioned above is that they only consider a single objective. 

However, managers are usually faced with complex multiobjective optimization problems (MOPs) in reality. For the JRD policy, it is necessary to decrease the total cost while improving the service level. Although there are several papers that studied multiobjective inventory models (Roy and Maiti [16]; Rong et al. [17]; Islam [18]; Wee et al. [19]), no study on the multiobjective JRD can be found.

For MOPs, direct comparison among the solutions is very difficult because of the different measurements between each contradicted target. In this study, total cost and service level are obviously two contradictory targets: reducing total cost may result in the decline of service level and vice verse. So we should coordinate two targets. Different from a single-objective optimization problem which has unique optimal solution, an MOP has a set of optimal solutions called Pareto optimal solutions. Due to the characteristics of MOPs, they are much more complex and the key is to find an effective method to obtain Pareto optimal solutions. Unfortunately, the classical JRPs and JRDs are already NP hard problems (Arkin et al. [20]), and the multiobjective makes the JRDs become much more difficult to handle. 

Many linear or nonlinear weighted methods (Rong et al. [17]; Islam [18]; Wee et al. [19]; Roy and Maiti [16]) were used to convert the multiobjective to a single one in the existing studies. These methods undoubtedly provide one easy way to deal with the multiobjective JRD model. However, these approaches do not solve the MOPs intrinsically, since the solutions for MOPs are multiple rather than one. On the other hand, multiobjective optimization methods based on Pareto-based MOEAs are widely used, such as multiobjective genetic algorithm (MOGA) (Aiello et al. [21]), nondominated sorting genetic algorithm (NSGA) (Lin [22]), and strength Pareto evolutionary algorithm (SPEA) (Zitzler and Thiele [23]; Sheng et al. [24]).

In recent years, several MOEAs based on Pareto differential evolution (DE) were utilized to solve MOPs. Santana-Quintero and Coello [25] presented a DE-based multiobjective algorithm using a secondary population and the concept of *ϵ*-dominance. The performance of the proposed algorithm was also compared with NSGA-II and *ϵ*-MOEA. Qian et al. [26] proposed a memetic algorithm based on DE (MODEMA) for multiobjective job shop scheduling problems. Qian and li [27] proposed an adaptive DE (ADEA) and the results of five test functions showed that the ADEA was very efficient to find out the true Pareto front. However, the majority of the above studies focused on the effectiveness verification of the algorithms and always analyzed standard testing functions and ignored the practical applications of them in MOPs. Due to the existing unpredictable and uncertain factors of inventory management, it is difficult to convert shortage rate/quantity to shortage cost. Therefore, discussing shortage rate/quantity independently is meaningful. 

The aim of this study is to propose a new multiobjective stochastic JRD (MSJRD) model including two minimum objectives, that is, the total cost and shortage quantity. The main difference between this study and [10] is that two objectives are handled simultaneously. Moreover, effective approaches are provided to handle this MSJRD. Having the successful applications in engineering management, as well as the effectiveness of DE in solving MOPs and JRPs/JRDs (Wang et al. [28–30]), linear programming (LP) and MOEA-based approaches using DE are provided. Results of an example show that the proposed hybrid DE is more effective than the original DE and GA whatever LP or MOEA method is used.

The rest of this paper is organized as follows.  Section 2 describes the proposed multiobjective stochastic JRD model.  Section 3 introduces the hybrid DE.  Section 4 presents two approaches to solve the MSJRD.  Section 5 contains numerical examples and results.  Section 6 discusses conclusions and provides future research directions.

## 2. Formulation of the Proposed MSJRD Model

Consider an enterprise has a center warehouse in a proper place and several suppliers in decentralized locations. The center warehouse jointly replenishes items from its suppliers according to the market demand or historical data. Then, the center warehouse will collect replenished items from suppliers. In this situation, the center warehouse can jointly determine the replenishment and distribution policy to obtain the optimal decision.

Refering to the model of Qu et al. [9], the differences between the JRD policy and typical JRP can be concluded as follows: (a) the JRP supposes deterministic demand, while our model considers stochastic demand and allows for shortage; (b) the JRP just discusses the replenishment policy, while our model analyses not only replenishment but also transportation decision; (c) the JRP is a single objective model, while our model is a multiobjective model. 

For the MSJRD, reducing the related total cost as well as decreasing shortage quantity should be considered simultaneously. Therefore, the first target is to minimize total cost which consists of replenishment cost, inventory holding cost, and distribution cost. Minimizing the shortage quantity is the second target.

### 2.1. The First Target: Total Cost

The total cost includes inventory holding cost, replenishment cost, and distribution cost. Distribution cost involves the stopover cost at suppliers and cost related to distance. The infinite capacity of vehicle assumption makes the distribution problem a traveling salesman problem (TSP).

Inventory cost can be given as

(1) $$\begin{array}{c} \;C_{H}=\overset{n}{\underset{i=1}{\sum }}h_{i}\left[\frac{1}{2}D_{i}k_{i}T+z_{i}\sigma_{i}\sqrt{\left(L+k_{i}T\right)}\right]. \end{array}$$

In (1), the first term is deterministic inventory and the second one is safety stock. The following replenishment cost is the same as the classical JRP:

(2) $$\begin{array}{c} C_{S}=\frac{S}{T}+\overset{n}{\underset{i=1}{\sum }}\frac{s_{i}}{k_{i}T}=\frac{1}{T}\left(S+\overset{n}{\underset{i=1}{\sum }}\frac{s_{i}}{k_{i}}\;\right). \end{array}$$

When *k*_*i*_ are given, taking the least common multiple of *k*_*i*_ we can get integer *M*. It means that the replenishment and distribution behavior will be repeated every *M* period. For example, if *k*_*i*_ = (4,2, 1,1), then *M* = 4. That is to say, in period 1, all items should be replenished; in period 2, items 3 and 4 need to be replenished; in period 3, items 2, 3, and 4 should be replenished; in period 4, items 3 and 4 should be replenished. It is obvious that in period 5 the situation is the same as in period 1. The cycle period is *M*. Therefore, a limited horizon with *M* periods is used to calculate annual distribution cost.

Jointly replenishment of items can take the advantage of scale economies not only in the replenishment but also in distribution process. Four items specifically should be replenished in period 1 and it is advisable for the center warehouse to traverse suppliers that supply these items at one time. The shortest path in each period is considered so distribution cost can be obtained by solving a travelling salesman problem (TSP). The distribution cost is

(3) $$\begin{array}{c} C_{T}=\frac{\sum_{j=1}^{M}\left(\sum_{p=1}^{P}x_{pj}F_{p}+cd\left(j\right)\right)}{MT}, \end{array}$$

where $x_{pj}=\{\begin{array}{cc} 1, & \text{if}\;\text{supplier}\;p\;\;\text{is}\;\text{visited}\\ 0, & \text{otherwise}.\;\;\;\;\;\;\; \end{array}$

Therefore, the first target can be summarized as follows:

(4) F1(T,ki,zi)=CH+CS+CT=∑i=1nhi[12DikiT+ziσi(L+kiT)] +1T(S+∑i=1nsiki) +∑j=1M(∑p=1PxpjFp+cd(j))MT.

### 2.2. The Second Target: Stock-Out Quantity

In this study, the demand is assumed to follow normal distribution at interval of unit time, which is widely used in the literature (see Qu et al. [9]; Eynan and Kropp [31, 32]). This assumption means, that given *k*_*i*_ and *T* for item *i*, the demand will follow normal distribution over the interval of length *L* + *k*_*i*_ *T* with the expectation *E* = *D*_*i*_(*L* + *k*_*i*_ *T*), variance Var⁡ = *σ*_*i*_^2^(*L* + *k*_*i*_ *T*) and probability density function *f*(*x*_*i*_, *L* + *k*_*i*_ *T*).

With a periodic replenishment policy, stockout could occur any time during replenishment intervals as long as the real demand exceeds maximum inventory level *R*_*i*_. The total annual stock-out quantity is

(5) F2(T,ki,zi)=∑i=1n(∫Ri∞(xi−Ri)f(xi,L+kiT)dxi)kiT=∑i=1nσikiTkiT+L∫zi∞(y−zi)f(y)dy=∑i=1nσikiTkiT+L  ×(∫zi∞yf(y)dy−zi∫zi∞f(y)dy)=∑i=1nσikiTkiT+L(f(zi)−zi[1−F(zi)]),

where $R_{i}=D_{i}\left(k_{i}T+L\right)+z_{i}\sigma_{i}\sqrt{\left(k_{i}T+L\right)}$.

### 2.3. The MSJRD Model

The whole multiobjective model can be written as

(6) Min⁡ F1(T,ki,zi)=∑i=1nhi[12DikiT+ziσi(L+kiT)]  +1T(S+g(ki)+∑i=1nsiki),Min⁡ F2(T,ki,zi)=∑i=1nσikiTkiT+L ×(f(zi)−zi[1−F(zi)]),

where *g*(*k*_*i*_) = ∑_*j*=1_^*M*^(∑_*p*=1_^*P*^ *x*_*pj*_ *F*_*p*_ + *cd*(*j*))/*M*.

The goal of this multiobjective model is to find out the optimal *k*_*i*_, *z*_*i*_, and *T* to simultaneously minimize the total cost and stock-out quantity and thus to achieve Pareto solutions in which two objectives can be balanced. Two targets have different units of measurements and it is usually difficult to convert the shortage quantity to stock-out cost. In addition, they are often in conflict with each other, that is, decreasing shortage quantity may result in cost increasing.

MOPs are much more complex but closer to reality. Several traditional mathematic methods are used for solving multiobjective models, such as linear programming, goal programming, and analytic hierarchy process. However, they are successful only in small scale problems. Mathematic methods are too complex and too time consuming to solve large scale problems. In the following, we provide two common approaches based on an HDE to deal with the proposed MSJRD. Then a numerical example and comparative study between the proposed LP and MOEA are presented.

## 3. The Hybrid Differential Evolution Algorithm (HDE) 

### 3.1. The Classical DE

DE has been described as an effective and robust method to optimize some well-known nonlinear, nondifferentiable, and nonconvex functions. Due to its easy implementation, quick convergence, and robustness, DE has turned to be one of the best evolutionary algorithms in a variety of fields (Wang et al. [33]; Cui et al. [34]). DE contains three operations: mutation, crossover, and selection.

#### 3.1.1. Mutation

The mutation operation creates a new vector by adding the weighted difference of two random vectors to a third one. For each target vector *x*_*t*_^*G*^  (*t* = 1, 2 … NP), the mutated vector is created as follows:

(7) $$\begin{array}{c} v_{t}^{G+1}=x_{r_{1}}^{G}+F\times \left(x_{r_{2}}^{G}-x_{r_{3}}^{G}\right). \end{array}$$

In (7), *r*_1_, *r*_2_, and *r*_3_, are three serial numbers of vectors, which are randomly generated with different values and none of them equals *t*. Three vectors *x*_*r*_1__^*G*^, *x*_*r*_2__^*G*^, and *x*_*r*_3__^*G*^ will be selected from the population for mutation operation when *r*_1_, *r*_2_, and *r*_3_ are confirmed, *F* is a scaling factor and *G* is the current number of iteration. 

#### 3.1.2. Crossover

A trail vector is created by mixing the mutated vector with the target vector according to the following formula:

(8) utjG+1={vtjG+1,if randm(j)≤CR  or  j=randn(t)xtjG,otherwise,

where *j* represents the *j*th dimension; *randm*(*j*) is randomly generated from 0 to 1; *randn*(*t*)∈[1,2,…, *D*] is a randomly selected integer to ensure the effect of mutated vector; CR is the crossover probability and it is very important for DE since the larger CR is, the more *v*_*t*_^*G*+1^contributes to *u*_*t*_^*G*+1^. 

#### 3.1.3. Selection

The selection operation is implemented by comparing the trial vector (obtained through mutation and crossover operations) with the corresponding target vector. For example, to minimize the function, the next generation is formed by

(9) xtG+1={utG+1,if  f(utG+1)<f(xtG),xtG,otherwise,

where the *f*( · ) is the fitness function of DE. 

Here, an example is given to illustrate the three operations mentioned previously. For the current number of iteration *G* and target vector *x*_1_^*G*^, suppose that random generated numbers *r*_1_, *r*_2_, and *r*_3_ are 23, 40, and NP respectively, and we obtain the following:

(10) Target  vectors  xG:Vector  x1:65240.090⋯Vector  x23:  54210.578⋯Vector  x40:  51360.745⋯Vector  xNP:  42570.024.

Mutation: if *F* = 0.6, the mutated vector *v*_1_^*G*+1^ can be obtained by (7) as follows:

(11) Mutated  vectors  vG+1:Vector  v1:5.63.40.80.41.010⋯

Crossover: if CR = 0.3, *randn*(*t*) = 3 (here *t* = 1), and vector *randm* = (0.1, 0.4, 0.5, 0.2, 0.6), the trial vector can be obtained by (8) as follows:

(12) Trial  vectors  uG+1:Vector  u1:5.650.80.40.090⋯

Selection: then target *x*_1_ should be compared with *u*_1_. Since *f*(*u*_1_^*G*+1^) < *f*(*x*_1_^*G*^), vector *u*_1_ should be selected to the next generation as follows:

(13) Next  generation  xG+1:Vector  1:5.650.8  0.40.090⋯

### 3.2. The Proposed Hybrid DE (HDE)

The typical DE is simple and easy to be implemented. However, it is likely to be premature too early. One-to-one competing is one of the main reasons. Therefore, improvements including dynamic parameter adjusting, different mutation and crossover strategies, or hybrid algorithms are necessary to be adopted.

Similar to DE, a genetic algorithm (GA) contains crossover, mutation, and selection operations. The crossover operation of GA is quite complicated and its complexity may grow rapidly when the problem scale becomes larger. Fortunately, GA has several efficient selection operations such as roulette wheel selection, tournament selection, and truncation selection. In this study, an HDE that combines the advantages of DE and GA is proposed. The proposed HDE can simplify the evolutionary process, and it can overcome the limitation of one-to-one selection of DE and thus prevent premature convergence.

**Figure 1:**
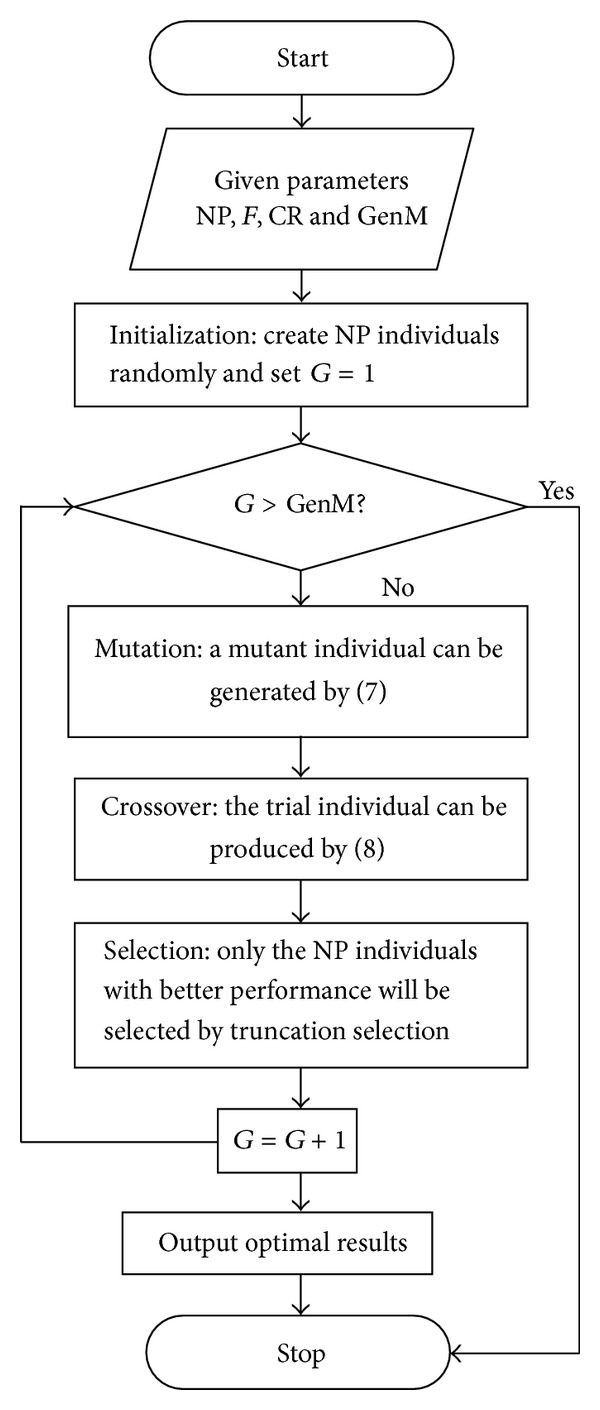
Flow chart of HDE.

Actually, several scholars also proposed hybrid DEs based on DE and GA (Hrstka and Kučerová [35]; He et al. [36]; Lin [37]), but their mixing modes are quite different from ours. In the proposed HDE, the mutation and crossover operations are the same as in DE while the selection operation is from truncation selection of GA. That is to say, it will be reserved instead of comparing with the target vector when a trial vector is generated. When all trail and target vectors are determined, top NP vectors with better performance are selected to the next generation. The HDE-based procedure is shown in Figure 1. 

## 4. Two Methods for Solving MSJRD

### 4.1. Linear Programming (LP) Approach for the MSJRD

This method is to summarize the weighted targets and thus converts the multiobjective model to a single one. Take into consideration that two targets have different measurements; it is necessary to standardize two targets beforehand.

#### 4.1.1. Model Analysis Using Linear Programming Approach

Suppose that the weights of two objectives are *w*_1_ and *w*_2_, and then the multiobjective problem can be described as

(14) Max⁡ λ=w1u1+w2u2s.t. {u1=F1max⁡(T,ki,zi)−F1(T,ki,zi)F1max⁡(T,ki,zi)−F1min⁡(T,ki,zi)u2=F2max⁡(T,ki,zi)−F2(T,ki,zi)F2max⁡(T,ki,zi)−F2min⁡(T,ki,zi).w1+w2=1,

*F*_1_^max⁡^(*T*, *k*_*i*_, *z*_*i*_),  *F*_1_^min⁡^(*T*, *k*_*i*_, *z*_*i*_), *F*_2_^max⁡^(*T*, *k*_*i*_, *z*_*i*_), and *F*_2_^min⁡^(*T*, *k*_*i*_, *z*_*i*_) are the tolerant maximum total cost, minimum total cost, maximum stock-out quantity, and minimum stock-out quantity, respectively, which can be given in advance by decision makers (Wee et al. [18]).

Set *w*_1_′ = *w*_1_/(*F*_1_^max⁡^ − *F*_1_^min⁡^), *w*_2_′ = *w*_2_/(*F*_2_^max⁡^ − *F*_2_^min⁡^). Then, the objective function is changed to

(15) Max⁡ λ=Fc−(w1′F1+w2′F2),

where *F*_*c*_ = *w*_1_ *F*_1_^max⁡^/(*F*_1_^max⁡^ − *F*_1_^min⁡^) + *w*_2_ *F*_2_^max⁡^/(*F*_2_^max⁡^ − *F*_2_^min⁡^) and

(16) Fw=w1′F1+w2′F2=w1′(∑i=1nhi[12DikiT+ziσi(L+kiT)]+1T[S+g(ki)+∑i=1nsiki]) +w2′(∑i=1nσikiTkiT+L(f(zi)−zi[1−F(zi)])).

Let ∂*F*_*w*_/∂*z*_*i*_ = 0, that is, $w_{1}^{\prime }h_{i}\sigma_{i}\sqrt{k_{i}T+L}+w_{2}^{\prime }{(\sigma }_{i}/k_{i}T)\sqrt{k_{i}T+L}\left(\left(df(z_{i})/{dz}_{i})-[1-F(z_{i})]+z_{i}f(z_{i}\right)\right)=0$.

Note that for standard normal distribution, *df*(*z*_*i*_)/*dz*_*i*_ = −*z*_*i*_ *f*(*z*_*i*_), so that

(17) $$\begin{array}{c} 1-F\left[z_{i}\left(k_{i}T\right)\right]=\frac{w_{1}^{\prime }h_{i}}{w_{2}^{\prime }}k_{i}T. \end{array}$$

That is to say, when *k*_*i*_ and *T* is known, the optimal value of *z*_*i*_ must satisfy (17).

Taking the second derivation of *TC*(*T*, *k*_*i*_, *z*_*i*_) with respect to *z*_*i*_, we obtain $\partial^{2}TC(T,k_{i},z_{i})/{\partial z}_{i}^{2}=(w_{2}^{\prime }\sigma_{i}/k_{i}T)\sqrt{k_{i}T+L}f(z_{i})>0$, which means that the optimal *z*_*i*_ is derived from (17) and *z*_i_*(*k*_*i*_ *T*) = *F* ^−1^(1 − (*w*_1_′*h*_*i*_/*w*_2_′)*k*_*i*_ *T*).

Substituting *z*_i_*(*k*_*i*_ *T*) into (16), the optimal value of *F*_*w*_ for given *k*_*i*_ and *T* is

(18) Fw=w1′(∑i=1nDihiki2T+1T[S+g(ki)+∑i=1nsiki]) +w2′(∑i=1nσikiTkiT+Lf(zi∗)).

Finally, the linear programming model can be written as

(19) Max⁡ λ(T,ki)=Fc−w1′(∑i=1nDihiki2T+1T[S+g(ki)+∑i=1nsiki])     −w2′(∑i=1nσikiTkiT+Lf(z  i∗))s.t. {Fc=w1F1max⁡F1max⁡−F1min⁡+w2F2max⁡F2max⁡−F2min⁡,w1′=w1F1max⁡−F1min⁡,w2′=w2F2max⁡−F2min⁡,w1+w2=1z  i∗(kiT)=F−1(1−w1′hiw2′kiT).

The goal is to determine the best *k*_*i*_ and *T* to maximize *λ* for the given *w*_1_ and *w*_1_.

#### 4.1.2. HDE-Based Procedures for MSJRD Using LP Approach

When *k*_*i*_ are determined, the optimal delivery cost can be calculated by solving a TSP. *z*_i_*(*k*_*i*_ *T*) can be calculated by *z*_i_*(*k*_*i*_ *T*) = *F* ^−1^(1 − (*w*_1_′*h*_*i*_/*w*_2_′)*k*_*i*_ *T*) when *k*_*i*_ and *T* are known. Change *k*_*i*_ and *T* with the following steps until maximum *λ* is obtained.

**Step 1**

Initialization: set related parameters (CR, F, and NP) for HDE. Set the lower bound (*k*_*i*_^LB^) and the upper bound (*k*_*i*_^UB^) of *k*_*i*_ respectively. Note that *k*_*i*_ are integers so, *k*_*i*_^LB^  is obviously 1. According to experience of [2, 10, 15], *k*_*i*_^UB^  is set sufficiently large to guarantee that the optimal solution does not escape. In this study, it can be set to 100. *T* is randomly generated in the range of 0 and 1. Combining *k*_*i*_ and *T* we get the *t*th individual *x*_*t*_ = (*k*_*i*_, *T*). Create initial population randomly.

**Step 2**

For a given *w*_1_, calculate the objective function. When *x*_*t*_ is determined, *z*_i_*(*k*_*i*_ *T*) can be derived accordingly. *x*_*t*_  and *z*_i_*(*k*_*i*_ *T*) jointly determine *λ*.

**Step 3**

Differential operations: while stopping criterion is not met, implement mutation and crossover for each individual. After that, the number of population is two times the original one.

**Step 4**

Genetic operations: select the individuals according to *λ*. Those with larger *λ* will be chosen to the next generation. Then the number of population is the same as the original one.

**Step 5**

When the number of iteration reaches a predefined maximum number, output the optimal results; otherwise, repeat Steps 2–4.

### 4.2. Multiobjective Evolution Algorithm (MOEA) Approach for the MSJRD

In this section, a brief introduction of MOP is given. Then, an HDE-based procedure to handle the MSJRD using noninferior and crowding distance is designed.

#### 4.2.1. Some Definitions of MOP

**Definition 1 (multiobjective optimization problems (MOP))**

(20) Min⁡F(x)=f1(x),f2(x),…,fk(x)Subject  to: gi(x)≤0, i=1,2,…,m.

A general MOP consists of *n* decision variables, *k* objective functions, and *m* constrains. In Definition 1, *x* refers to the decision space and *g*_*i*_(*x*) are constrains of MOP.

**Definition 2 (Pareto optimal solution)**

The  optimal solution of MOP is often referred to as the Pareto optimal solution. Let vector *a* belong to *x* and suppose that x* is a subset of *x*. If there does not exist any vector in x* that is better than *a*, then *a* is called noninferior solution (or Pareto optimal solution) of x*. Moreover, if vector *a* is the noninferior solution of *x*, then vector *a* is the Pareto optimal of the MOP.

#### 4.2.2. HDE-Based Procedures for MSJRD Using MOEA Approach

There exist many difficulties when applying DE to solve an MOP compared with single objective problem. The main challenges for solving MOP are as follows: how to generate offspring and how to keep Pareto solutions uniformly distributed. The classical DE is not suitable for an MOP since many good solutions may be abandoned due to its one-to-one competing mechanism. This will also be confirmed by a numerical example. 

Therefore, we also use an HDE which uses truncation selection to choose next generation based on front rank and crowding distance adopted by Qian and li [27]. The steps of calculating crowding distance are presented in Algorithm 1.

In this algorithm, the low front rank corresponds to the high quality of a solution. As to the those individuals with the same front rank, the larger crowding distance means better distribution. Therefore, individuals with lower front rank and larger crowding distance are selected to the next generation.

The first target can be divided into an inventory problem and a delivery problem. When all *k*_*i*_ are determined, the optimal delivery cost can be calculated by solving a TSP. In addition, for a stochastic JRP with normal distributed demand, when *k*_*i*_, *z*_*i*_, and *T* are known, the stochastic JRP can then be solved. With the same value of *k*_*i*_, *z*_*i*_, and *T* in the second target, we can obtain the corresponding value of the second target. Then change *k*_*i*_, *z*_*i*_, and *T* with the following steps until the termination condition is satisfied. The steps of HDE-based approach are described as follows.

**Step 1**

Initialization: set related parameters (CR, F, and NP) for the HDE. Set the lower bound and the upper bound of *k*_*i*_, respectively; that is, *k*_*i*_^LB^ = 1 and *k*_*ij*_^UB^ = 100. *z*_*i*_ is randomly generated in the range of 0 and 3, which can cover 99.7% of the demand. *T* is randomly generated in the range of 0 and 1. Combining *k*_*i*_, *z*_*i*_, and *T* we get the *t*th individual *x*_*t*_ = (*k*_*i*_, *z*_*i*_, *T*). Create initial population randomly.

**Step 2**

Calculate the objective function, that is, the total cost and the total shortage quantity of all items.

**Step 3**

Calculate the Pareto front and crowding distance of each individual.

**Step 4**

Differential operations: while stopping criterion is not met, implement mutation and crossover for each individual. After that, the number of population is two times the original one.

**Step 5**

Genetic operations: select the individuals according to the front rank and crowding distance. Then the number of population is the same as the original one.

**Step 6**

When the number of iteration reaches a predefined maximum number, output the optimal results; otherwise, repeat Steps 2–5.

## 5. Contrastive Example and Results Analysis 

### 5.1. Basic Data of Numerical Example

The data come from Qu et al. [9].  Table 1 describes the items to be replenished and the center warehouse correspondingly. Tables 2 and 3 are the related parameters of items and distances between suppliers and warehouse, respectively.

In the following, two approaches named LP and MOEA are compared. The comparison contains two aspects: the Pareto solutions and some specific solutions obtained by each method. In the meanwhile, three algorithms used in each method are compared with each other.  Table 4 reports related parameters of HDE, DE, and GA.

For LP-based approach, we directly set *F*_1_^max⁡^(*T*, *k*_*i*_, *z*_*i*_) = 10500, *F*_1_^min⁡^(*T*, *k*_*i*_, *z*_*i*_) = 7500, *F*_2_^max⁡^(*T*, *k*_*i*_, *z*_*i*_) = 120, and *F*_2_^min⁡^(*T*, *k*_*i*_, *z*_*i*_) = 0 according to the advice of the decision makers. This approach is also widely used by other scholars (Wee et al. [18]).

### 5.2. Comparisons for LP-Based and MOEA-Based Solutions

In this section, the above numerical example is handled using LP and MOEA. For LP, the weight of each objective must be assigned firstly. In order to compare with MOEA, the objectives can be converted to single index by setting the total cost and total shortage quantity with the same weights for MOEA when the Pareto solutions are obtained. The best results for LP when *w*_1_ = 0.56 are presented in Table 5. As to MOEA, the highest index after converting is shown in Table 6.

Table 5 shows that HDE and DE are better than GA for LP; Table 6 implies that HDE is better than GA and GA is better than DE for MOEA. In order to further verify the conclusion, we obtained for different *w*_1_, *w*_1_is set from 0.1 to 0.9 and the results are reported in Table 7.

Set the total cost and total shortage quantity with the same weights *w*_1_ and *w*_2_, respectively, for MOEA. Then, solutions with the highest weighted objective from the obtained Pareto solutions are shown in Table 8. 

Table 7 shows that values of each target *F*_1_ and *F*_2_ change correspondently when *w*_1_ varies, which means that different settings of the weight will result in different decisions. Moreover, when the weight of the first objective (total cost) equals 0.1, the solution is the best. When the difference between *w*_1_ and *w*_2_ becomes smaller, the solutions become worse. Table 8 implies a similar conclusion for HDE, DE, and GA.

From the comparisons for specific solutions, the following conclusion can be easily drawn: (1) HDE is better than DE or GA no matter whether LP or MOEA is adopted: HDE and DE are more suitable than GA when LP is used; HDE and GA are better than DE when MOEA is used. (2) Different weights for objectives will influence the solutions, for the conflicted objectives, and the assigned weights with large ratios (i.e., *w*_1_ : *w*_2_ ≥ 3 : 1) may result in better solutions.

### 5.3. Nondominated Solution Analysis of MOEA

For the MOP, there have several metrics to evaluate the quality of the nondominated solutions (Robič and Filipič [38]). However, the implementation of most metrics needs a prerequisite; that is, the true Pareto front must be known. In this study, it is impossible to find the true Pareto front because the MSJRD is a practical problem. So we adopt the metric (*Spacing*, *SP*) used by Esparcia-Alcázar et al. [39, 40] to measure the distribution of solutions on the Pareto front by evaluating the variance of neighboring solutions. The lower value of SP means that better nondominated solution is obtained.

SP measures the relative distances between the members of Pareto front as

(21) $$\begin{array}{c} \text{SP}=\sqrt{\frac{\sum_{i=1}^{n}{\left(\overset{-}{d}-d_{i}\right)}^{2}}{\left(n-1\right)}}, \end{array}$$

where *n* is the number of the first nondominated solutions found. The distance *d*_*i*_ is given by

(22) di=minj(|f1i(x)−f1j(x)|+|f2i(x)−f2j(x)|),  i,j=1,...,n,

where *f*_*N*_^*k*^(·) is the fitness of point *k* on objective *N* and $\overset{-}{d}$ is the mean of all *d*_*i*_.  Table 9 shows the mean and variance of SP by 10 runs using MOEA. 

Table 9 shows that the mean and variance of SP obtained by HDE is the lowest, and corresponding values obtained by DE are biggest. That is to say, HDE is better than DE and GA for the MOEA method. The conclusion is consistent with Section 5.2. At the same time, it verifies that the conversion using weights for the MSJRD is feasible.

**Figure 2:**
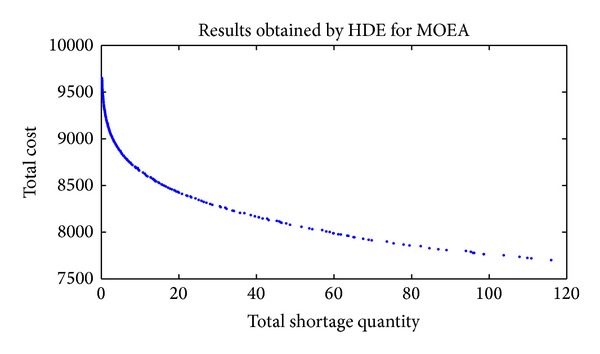
Nondominated solutions of the final population obtained by HDE.

In order to have a better understanding of the solutions' distribution of the last generation, the entire nondominated fronts found by HDE, DE, and GA are presented from Figure 2 to Figure 4.

**Figure 3:**
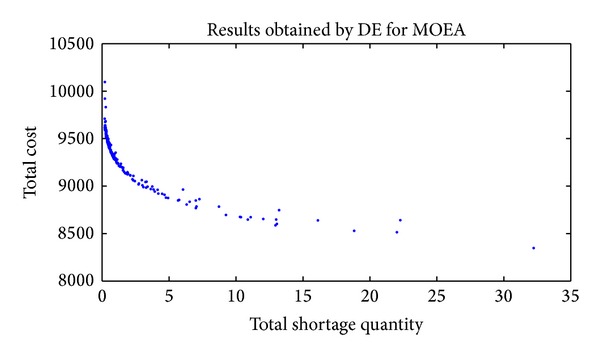
Nondominated solutions of the final population obtained by DE.

**Figure 4:**
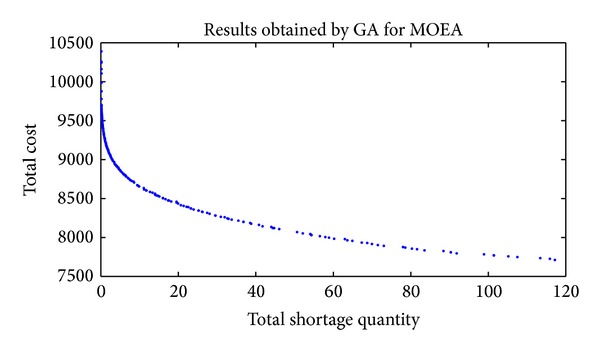
Nondominated solutions of the final population obtained by GA.

Figures 2, 3, and 4 show that HDE and GA are capable of obtaining Pareto solutions, while the effectiveness and distribution of solutions are much worse for DE. The main reason is the one-to-one competing of DE. When trial individual dominates target individual, the trial vector (otherwise the target vector) remains to the next generation. This mechanism is different from the selection operation of the HDE, where all the target and trial individuals are kept to be chosen according to the front rank and crowding distance. So, DE is not suitable for solving this MSJRD.

## 6. Conclusions and Future Research

In this study, a new multiobjective JRD model with stochastic demand is proposed which takes into account the service level while making the replenishment and delivery decisions. Then, two approaches to solve this complex optimization problem are designed using an improved HDE. The main contributions are as follows.

1. Considering the difficulty to estimate the shortage cost in reality, the shortage quantity is utilized as another standard to evaluate the rationality of decisions besides the total cost. To our best knowledge, this is the first time to propose a practical multiobjective stochastic JRD model.
2. The MOEA is adopted to solve the proposed multiobjective JRD model. The results of the numerical example and Pareto solution analysis show the feasibility of the MOEA to handle the proposed MSJRD. It enriches the application field of the MOEA.
3. The comparison of two approaches for the MSJRD verifies that LP and MOEA are suitable for solving this MSJRD problem. Furthermore, results show that the proposed HDE is more effective than DE and GA whatever LP or MOEA method is used. This illustrates that DE is likely to combine with other algorithms so as to provide a more effective way to solve complex problems.

The future research on the multiobjective JRD problem should consider more realistic assumptions such as uncertain costs, freight consolidation, and budget constraint.

## Figures and Tables

**Algorithm 1:**
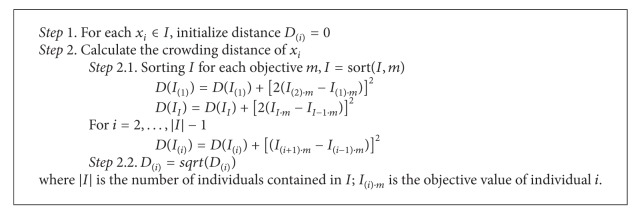
Steps of calculating crowding distance.

**Table 1:** Supply relationship between items and suppliers.

	Supplier 1	Supplier 2	Supplier 3
Item 1	1	0	0
Item 2	0	1	0
Item 3	0	0	1
Item 4	0	0	1
*F*_*p*_	40	50	60

**Table 2:** Parameters of items.

	Item 1	Item 2	Item 3	Item 4
*D* (unit/year)	600	900	1200	1000
*σ* ^2^ (unit^2^/year)	800	600	700	500
*L* (year)	0.02	0.02	0.02	0.02
*h* ($/unit/year)	5.6	21	42	15
*s* ($/order)	25	14	20	30
*π* ($/unit)	28	35	40	30

The other two parameters are as follows: *S* = 100 and *c* = 0.5.

**Table 3:** Distances between suppliers and warehouse.

	Warehouse	Supplier 1	Supplier 2	Supplier 3
Warehouse	0	11	9	7
Supplier 1	11	0	5	8
Supplier 2	9	5	0	10
Supplier 3	7	8	10	0

**Table 4:** Parameters of the algorithms.

Parameter	Value	Algorithms
Number of population, NP	200	HDE, DE, and GA
Maximum number of iteration, GenM	300	HDE, DE, and GA
Mutation factor, *F*	0.6	HDE and DE
Crossover rate, CR	0.3	HDE and DE
Probability of mutation, *P*_*m*_	0.1	GA
Probability of crossover, *P*_*c*_	0.9	GA

**Table 5:** Results for LP with HDE, DE, and GA (*w*_1_ = 0.56).

	HDE	DE	GA
*k*	2, 1, 1, 1	2, 1, 1, 1	2, 1, 1, 1
*z*	1.67, 1.35, 0.93, 1.53	1.67, 1.35, 0.93, 1.53	1.70, 1.38, 0.96, 1.56
*T*	0.0824	0.0824	0.0785
*F*_1_	8468.21	8468.21	8487.52
*F*_2_	17.44	17.44	16.91
*λ*	0.7553	0.7553	0.7536

**Table 6:** Results for MOEA with HDE, DE, and GA (*w*_1_ = 0.56).

	HDE	DE	GA
*k*	2, 1, 1, 1	3, 1, 1, 1	2, 1, 1, 1
*z*	1.59, 1.35, 0.96, 1.49	1.49, 1.64, 1.11, 1.67	1.55, 1.37, 0.99, 1.46
*T*	0.0721	0.0736	0.0721
*F*_1_	8466.57	8587.38	8475.24
*F*_2_	17.70	12.95	17.36
*λ*	0.7547	0.7495	0.7543

**Table 7:** Results for LP with HDE, DE, and GA (*w*_1_ varies).

*w*_1_	HDE			DE			GA		
	*F*_1_	*F*_2_	*λ*	*F*_1_	*F*_2_	*λ*	*F*_1_	*F*_2_	*λ*
0.1	9253.01	1.02	0.9339	9256.99	1.01	0.9339	9252.98	1.02	0.9339
0.2	9031.90	2.55	0.8809	9040.17	2.48	0.8808	9117.39	2.40	0.8762
0.3	8867.45	4.74	0.8356	8867.69	4.76	0.8354	8873.75	4.67	0.8354
0.4	8718.56	7.96	0.7977	8716.80	8.06	0.7975	8760.01	7.59	0.7941
0.5	8567.08	12.96	0.7682	8574.14	12.77	0.7678	8591.70	12.51	0.7659
0.6	8396.13	21.43	0.7493	8400.40	21.34	0.7488	8409.68	20.93	0.7483
0.7	8176.59	38.11	0.7468	8183.03	37.86	0.7460	8172.24	38.64	0.7465
0.8	7890.98	72.37	0.7751	7892.54	72.87	0.7739	7894.30	72.40	0.7742
0.9	7674.80	123.83	0.8444	7674.80	123.83	0.8444	7656.65	130.97	0.8439

**Table 8:** Results for MOEA with HDE, DE, and GA (*w*_1_ varies).

*w*_1_	HDE			DE			GA		
	*F*_1_	*F*_2_	*λ*	*F*_1_	*F*_2_	*λ*	*F*_1_	*F*_2_	*λ*
0.1	9253.73	1.03	0.9338	9285.77	0.89	0.9337	9225.35	1.17	0.9337
0.2	9046.73	2.42	0.8807	9017.73	2.72	0.8806	9031.13	2.59	0.8807
0.3	8886.03	4.48	0.8352	8878.41	4.77	0.8343	8848.34	5.17	0.8350
0.4	8720.04	8.02	0.7972	8768.47	7.00	0.7959	8739.46	7.55	0.7970
0.5	8545.97	13.94	0.7676	8587.38	12.95	0.7648	8617.66	11.16	0.7672
0.6	8423.72	20.00	0.7486	8587.38	12.95	0.7394	8359.45	23.93	0.7483
0.7	8207.26	35.76	0.7456	8348.16	32.22	0.7215	8229.71	33.76	0.7453
0.8	7913.56	69.71	0.7735	8348.16	32.22	0.7201	7892.98	73.03	0.7735
0.9	7685.28	121.57	0.8431	8348.16	32.22	0.7187	7711.52	117.17	0.8389

**Table 9:** Statistical analysis of SP by 10 runs.

	Mean	Variance
HDE	8.75	1.93
DE	17.48	10.48
GA	12.15	3.96

## Abbreviations

*D*_*i*_: Annual demand rate of item *i*
*S*: Major ordering cost
*s*_*i*_: Minor ordering cost of item *i*
*h*_*i*_: Annual inventory holding cost of item *i*
*T*: Basic cycle time (decision variable)
*k*_*i*_: Multiplier of *T* (decision variable)
*T*_*i*_: Cycle time of item *i* (decision variable)
*R*_*i*_: Maximum inventory level of item *i* during replenishment interval
*z*_*i*_: Safety stock factor of item *i* (decision variable)
*L*: Lead time of items
*σ*_*i*_: Standard deviation of item *i*
*M*: Least common multiple of *k*_*i*_
*π*_*i*_: Stock-out cost per unit for item *i*
*c*: Distribution cost per unit distance
*F*_*p*_: Stopover cost at supplier *p*
*d*(*j*): Shortest path needed for distribution in *j*th basic cycle.
